# Common variable immunodeficiency complicated with hemolytic uremic syndrome

**DOI:** 10.3109/03009734.2011.635815

**Published:** 2012-02-15

**Authors:** Biljana Milošević, Vesna Stojanović, Marko Nikolić, Georgios Konstantinidis, Andrija Rudić

**Affiliations:** ^1^Department of Nephrology, Institute for Child and Youth Health Care of Vojvodina, Novi Sad, Serbia; ^2^Intensive Care Unit, Institute for Child and Youth Health Care of Vojvodina, Novi Sad, Serbia; ^3^Department of Immunology and Allergology, Institute for Child and Youth Health Care of Vojvodina, Novi Sad, Serbia

**Keywords:** Child, common variable immunodeficiency, hemolytic uremic syndrome

## Abstract

Common variable immunodeficiency is a primary immunodeficiency disease characterized by reduced serum immunoglobulins and heterogeneous clinical features. Recurrent pyogenic infections of upper and lower respiratory tracts are the main clinical manifestations of common variable immunodeficiency. Hemolytic uremic syndrome is a multisystemic disorder characterized by thrombocytopenia, microangiopathic hemolytic anemia, and organ ischemia due to platelet aggregation in the arterial microvasculature. This is one of the rare cases of patients diagnosed with common variable immunodeficiency, which was complicated by hemolytic uremic syndrome.

## Introduction

Common variable immunodeficiency (CVID) is a disorder characterized by decreased levels of serum immunoglobulins (IgG and IgA, and 50% of patients have diminished levels of IgM) and an increased susceptibility to infection (1). It is a common form of immunodeficiency with a variable clinical course. Most patients have sporadic disease, but in the past 5 years investigators have described defects in four genes associated with CVID (autosomal dominant or autosomal recessive inheritance). Park et al. have described genetic defects in a few patients with CVID, but nearly 75% of patients have no known defect (1,2). The prevalence of CVID is approximately 1:50,000–200,000, with a reported incidence of 1 per 75,000 live births (3,4). Recurrent pyogenic infections of upper and lower respiratory tracts are the main clinical manifestations of CVID. These patients are more likely to have increased frequencies of hepatosplenomegaly, iridocyclitis, autoimmune hemolytic anemia, immune thrombocytopenic purpura, malabsorption, inflammatory bowel disease, failure to thrive, and autoimmune disease (5,6).

The definition of CVID includes three key features: the presence of hypogammaglobulinemia of two or more immunoglobulin classes (low IgG, IgA, or IgM), recurrent sinopulmonary infections, and impaired functional antibody responses. The criteria for impaired functional antibody responses include absent isohemagglutinins, poor responses to protein (diphtheria, tetanus) or polysaccharide vaccines (*Streptococcus pneumoniae*), or both (1,3,7). Intravenous immunoglobulin (IVIG) is effective and is currently the mainstay of therapy for CVID. Intravenous immunoglobulins lower the incidence of pneumonic and recurrent bacterial infections, thus preventing chronic pulmonary disease (8–10). The prognosis for patients with CVID is reasonably good. Early diagnosis and therapy are essential in improving the outcome in patients with CVID (11,12).

Hemolytic uremic syndrome (HUS) is primarily a disease of infancy and early childhood and is typically characterized by the triad of microangiopathic hemolytic anemia, thrombocytopenia, and acute renal failure (13,14). Two predominant types of HUS are identified: one type involves diarrhea (D+) (95% of cases) and the other does not (D– or atypical). D+ HUS occurs predominantly in children and is preceded by a prodrome of diarrhea, most commonly caused by an infection by shiga-toxin-producing *Escherichia coli*. D– HUS accounts for the remaining 5% of cases of HUS, and its etiology, age at onset, and clinical presentations are far more varied. The pathogenesis of D– HUS has been the focus of current research and has, thus far, been associated with complement dysregulation in up to 50% of cases. Clinically, D– HUS has been associated with various non-enteric infections, viruses, drugs, malignancies, transplantation, pregnancy, and other underlying medical conditions such as scleroderma and antiphospholipid syndrome. Infections caused by *Streptococcus pneumoniae* have been linked to 40% of D– HUS cases (15). Incidence of D– HUS in children is approximately 2 cases per year per 100,000 total population (13). Plasma exchange (plasmapheresis combined with fresh-frozen plasma replacement) is currently the treatment of choice. Plasma exchange is performed daily until remission is obtained (16). Patients with D– HUS collectively have a poor prognosis, and as many as 50%–60% progress to end-stage renal disease or develop irreversible brain damage. About 25% die during the acute phase (17).

This paper reviews the case of a 5-year-old boy with CVID complicated with HUS.

## Case report

A boy, aged 5 years, had a bad cough with breathing difficulties a month before hospitalization and received out-patient treatment with antibiotics and bronchodilators. A week before hospitalization he was swollen in his face, he urinated less, and was extremely dyspneic. The boy was hospitalized at our institute, in the intensive care unit.

Since his early infancy, the boy had had repeated bacterial respiratory infections (adenoid, middle ear, obstructive bronchitis, and pneumonia), while at the age of 1 he was diagnosed with generalized lymphadenopathy and hepatosplenomegaly. During his second year of life, he underwent an adenoidectomy. Due to hepatosplenomegaly, the boy had been monitored by a hematologist between the ages of 2 and 4.5. Previous laboratory findings: Immunoglobulins of serum IgA 0.24 g/L (0.41–2.97), IgM 0.67 g/L (0.4–1.6), IgG 5.5 g/L (6–13). Alfa 1 antitrypsin was within the reference value. There were no indications of co-natal and acquired virus infections, hemoglobinopathy (fetal hemoglobin and haptoglobin within the reference values), malignant diseases (bone-marrow biopsy, myeloid hyperplasia). The pathohistological findings of a peripheral lymph gland sample showed reactive lymphadenitis. In the later course, pancytopenia was diagnosed. During the stated period, the clinical features were dominated by obstructive pulmonary disease, and a physical examination showed splenomegaly and generalized lymphadenopathy, accompanied by a failure to thrive (the boy had very poor appetite, especially during respiratory infections which he frequently had). The mental status was preserved. There were no similar diseases in the family.

At admission, the boy was in a generally difficult condition, afebrile, with tachycardia (140/min), tachydyspnea (50/min), blood pressure 120/80 mmHg. His body weight was 17 kg (below third percentile) and his height 101 cm (below third percentile). The skin and the visible mucous membranes were pale, acrocyanotic, and edematous. The lymph glands were swollen, approximately 2 cm in diameter. Pulmonary auscultation revealed inspiratory crackles, early and late, with low-pitch wheezing. Heart action: gallop rhythm, no murmur, with weaker peripheral pulses. He had hepatosplenomegaly. Neurological findings were normal.

Laboratory findings were as follows: increased acute phase reactants (sedimentation rate, C-reactive protein), pancytopenia (leukocyte count 2.7 × 10^9^/L), thrombocyte count 40 × 10^9^/L; Coombs positive test, hemolytic anemia, Hb 64 g/L, and reticulocytes 1.2%. Osmotic resistance of red blood cells was normal. Peripheral blood smear showed presence of fragmented red blood cells. Serum creatinine, 153 μmol/L (estimated creatinine clearance 30 mL/min/1.73 m^2^), urea nitrogen, 13.0 mmol/L (0.1–7), acidum uricum, 278 μmol/L (71–230). Activated partial thromboplastin time (aPTT), prothrombin time (PT), D-dimer, fibrinogen: normal findings. Total serum proteins 55.5 g/L, albumin 24 g/L. Aspartate aminotransferase, alanine aminotransferase, gamma-glutamyltransferase, bilirubin: normal values. Cholesterol 7.28 mmol/L (up to 3), triglycerides 2.22 mmol/L (up to 0.7). Blood gas analysis indicated global respiratory insufficiency. Urine: protein 5+, microscopic hematuria. Hemoculture, stool culture, urinoculture: negative. Coxsackie virus, Cytomegalovirus, Hantaan virus (IgG and IgM): negative. Renal ultrasound: in both kidneys loss of corticomedullary differentiation, hyperechogenic parenchyma. Abdomen ultrasound: hepatosplenomegaly. Chest X-ray indicated inflammation and congestive changes with enlarged heart shadow. Echocardiography revealed left ventricular cardiomyopathy and pulmonary hypertension.

Immediately upon admission, mechanical ventilation with parenteral antibiotic therapy was administered (imipenem-cilastatin, clindamycin, sulfamethoxazole/trimethoprim, and fluconazole), followed by a conservative therapy for acute renal failure and acute pulmonary edema, and intravenous immunoglobulin (IVIG) therapy. On the third day of hospitalization the boy became comatose (Glasgow Coma Scale 4–7), with focal seizures. A lumbar puncture was performed to exclude infection of central nervous system (CNS) (the findings were normal), while computer tomography (CT) of the CNS revealed hyperdense changes sized 2 × 2 cm (intracerebral hematoma combined with ischemic lesions).

The diagnosed HUS (D– HUS; microangiopathic hemolytic anemia, thrombocytopenia and acute renal failure; the information about diarrhea was not received) was treated with repeated transfusions of fresh frozen plasma. The blood laboratory variables improved. The pulmonary function and consciousness were also improving; thus, on day 11, the boy was extubated and oxygen therapy was administered through a mask. As the acute renal insufficiency progressed (creatinine 461 μmol/L, urea nitrogen 56.5 mmol/L, acidum uricum 1620 μmol/L, estimated creatinine clearance 10.7 mL/min), on day 13 of hospitalization, a continuous ambulatory peritoneal dialysis (CAPD) was started. This led to some improvement in renal function test results and further improvement of consciousness, as well as the general condition, so the boy was transferred to the nephrology ward. In order to arrive at a final diagnosis, tests were continued to determine immunological disorders—immunoglobulin deficiency and immunological response, systemic disease (due to purpura on the trunk, vasculitis; and sclerodermatous-like changes on the right gluteus, 5 × 10 cm, and on the right lower leg, 5 × 3 cm, which were not present initially): antinuclear antibodies (ANA), anti-double stranded DNA, anti-RNP, anti-Sm, anti-SSA/Ro, anti-SS-B/La, anti-Scl-70, anti-Jo-1, anti-cardiolipin antibodies (ACA), antineutrophil cytoplasmic antibodies (ANCA), antimitochondrial antibodies, antiparietal antibodies, anti-smooth muscle antibodies, antiheart antibodies, and antithyroid antibodies: negative. Pathohistological examination of skin samples was non-specific and excluded scleroderma. IgA 0.16 g/L (0.41–2.97), IgG 3.3 g/L (6–13), IgM 0.13 g/L (0.4–1.6); all three classes of immunoglobulin were lowered in the three samples taken at monthly intervals. The antitetanus antibody titer was at the lower limit of the normal range. The nitroblue tetrazolium (NBT) test, opsonization, and bactericidal index were normal. Immunophenotyping: relative, absolute values of total T lymphocytes (CD3), as well as CD4 and CD8 subpopulation of T lymphocytes, were within physiological limits, with a preserved ratio (index CD4/CD8). Relative and absolute values of B lymphocytes (CD19), as well as NK cells (CD56), were within physiological limits. Immune complexes (PEG-198) and complement C1q, C3, and C4 had normal values. Blastic transformation of lymphocytes in culture stimulated by phytohemagglutinin (PHA) showed high spontaneous activity. Isohemagglutinins were negative.

Duodenal juice was free of *Giardia lamblia*. A microscopic stool examination revealed cysts of non-pathogenic ameba. A sweat chloride test was negative. Siderophages in bronchial aspirate was negative. CT scan of chest and abdomen showed enlarged thymus and mediastinal and retroperitoneal glands.

When suspicion of CVID was raised, a substitution therapy was introduced: IVIG (600 mg/kg per 3–4 weeks) with additional supportive therapy. Because autoimmune phenomena were present, he received corticosteroids during 1 month (prednisone 0.5 mg/kg). In the course of the therapy, the boy did not have respiratory infections; signs of chronic pulmonary disease were persistent; hemoglobin, leukocytes, and thrombocytes were normalized; and autoimmune phenomena were in complete regression. Consciousness and mental status were completely restored. A control CT scan of the CNS showed porencephalic changes in the place of the previously described lesions. After 3 months, peritoneal dialysis was discontinued, chronic renal insufficiency persisted (estimated creatinine clearance 35.0 mL/min/1.73 m^2^).

At the beginning, the diagnosis of CVID was difficult due to numerous complications and a slightly decreased level of immunoglobulin at the time of first medical check-up in the second year of life (levels of immunoglobulin in CVID may vary). During the first 3 months of treatment he was on renal replacement therapy (peritoneal dialysis) followed by 1 month of corticosteroid therapy, which also could lead to reduction in serum immunoglobulin levels. The cause of the loss of immunoglobulins, at the beginning of the disease, could be proteinuria, i.e. nephrotic syndrome as manifestation of HUS. However, in the further course, when he was not subjected to the above-described therapies, and when the proteinuria significantly decreased (to 470 mg/24 h; dipstick proteinuria test 2–3+), with the normalization of serum albumin, low levels of immunoglobulins still persisted (IgA 0.16, 0.36, and 0.2 g/L; IgM 0.13, 0.12, and 0.5 g/L; IgG 3.3, 3.0, and 2.9 g/L). Based on repeated analyses of immunoglobulin and the persistently low levels, the clinical features being frequent respiratory infections (bacterial, confirmed chronic pulmonary disease and bronchiectasis), growth retardation, failure to thrive, myocardiopathy, hepatosplenomegaly, autoimmune phenomena (similar to scleroderma), as well as negative isohemagglutinins and poor response to active immunization (tetanus toxoid), the diagnosis of CVID was established.

After 1 year, due to irregular substitution therapy by immunoglobulin, febrile respiratory infections recurred. As the chronic pulmonary disease progressed, the boy was on a home oxygen therapy. A year and a half after the basic disease had been diagnosed, the boy suddenly died during a bicycle ride, most probably due to cardiorespiratory insufficiency (the parents did not allow an autopsy).

## Discussion

CVID is one of the most prevalent primary immunodeficiency diseases. The age at presentation of CVID has a bimodal distribution. A few patients present in mid-childhood, but most present in early to mid-adulthood (1).

One-third of these patients develop diffuse or localized lymphadenopathy, intestinal nodular hyperplasia, or lymphoid infiltrates in the lung. Histologically, these infiltrates resemble the reactive lymphoid hyperplasia. The lungs are the most commonly affected site with multisystemic granulomas, though other organs, such as liver, skin, spleen, and gastrointestinal tract, can also be involved (18,19).

Some 25% of patients with CVID have autoimmune events—autoimmune thrombocytopenic purpura, autoimmune hemolytic anemia, pernicious anemia, autoimmune thyroiditis, rheumatoid arthritis, vitiligo, and vasculitis (1,6).

Chronic pulmonary complications, including recurrent pneumonia, are the primary causes of significant morbidity in patients with CVID. Pulmonary fibrosis and bronchiectasis occur frequently. In over 50% of patients with CVID chronic bronchitis and asthma are also recorded (20).

In the first place, the differential diagnosis included macrophage activation syndrome (MAS). It is characterized by uncontrolled hyperinflammation on the basis of inherited or acquired immune-mediated processes in which excessive activation and non-malignant proliferation of activated macrophages are observed. MAS can be included in the group of histiocytic disorders named ‘hemophagocytic lymphohistiocytosis’ (HLH). HLH can be primary or secondary. The secondary form can be caused by infection (mostly viruses, but also bacteria, protozoa, and fungi), malignancy (especially lymphomas), or autoimmune disease (MAS).

MAS is a severe, potentially life-threatening, complication of several chronic rheumatic diseases of childhood. It occurs most commonly with systemic-onset juvenile idiopathic arthritis (21,22). The diagnosis of MAS requires that five out of eight criteria be fulfilled. First, there are the five initial criteria: 1) fever; 2) cytopenia; 3) splenomegaly; 4) hypertriglyceridemia and/or hypofibrinogenemia; and 5) hemophagocytosis. Then there are three recent criteria: 6) low or absent NK cell activity; 7) hyperferritinemia; and 8) high plasma levels of soluble CD25 (23–25). First-line treatment for MAS is parenteral administration of high-dose corticosteroids. Steroid-resistant cases require the addition of cyclosporine A. Other therapeutic regimens are high-dose IVIG, antithymocyte globulins, etanercept, etoposide, and plasmapheresis (24).

In our patient, specified criteria such as cytopenia, splenomegaly, and hypertriglyceridemia were present (hypertriglyceridemia was understood as a manifestation of nephrotic syndrome within HUS; with the reduction of proteinuria and correction of serum albumin level, normalization of serum triglyceride level followed). Ferritin and plasma levels of soluble CD25, as well as bone-marrow biopsy, were not analysed due to technical reasons. Nevertheless, our patient fulfilled few clinical and laboratory criteria for the diagnosis of MAS. After the recovery from generalized bacterial infection, and courses of immunoglobulin replacement therapy, there was a rapid recovery of the boy’s general condition (he received short-term low-dose corticosteroid therapy when his general condition was already significantly improved). Retrospectively, such a clinical scenario favors our suspicion that it probably was CVID, not MAS.

At admission, the boy already had complications—chronic pulmonary disease with pulmonary hypertension, and consequential myocardiopathy, hepatosplenomegaly, lymphadenopathy, autoimmune phenomena, malabsorption, growth retardation, and failure to thrive. The likely diagnosis of CVID was based on the following criteria: frequent bacterial infections (consequently leading to advanced chronic pulmonary disease and bronchiectasis), growth retardation and failure to thrive, myocardiopathy, hepatomegaly, autoimmune phenomena (similar to scleroderma), decreased levels of IgG, IgA, and IgM, absence of isohemagglutinin synthesis, and poor response to active immunization (tetanus toxoid).

The heterogeneity in the clinical presentation of CVID presents a challenge to doctors. A definite diagnosis is often late because it is wrongly assumed that primary immunodeficiencies are extremely rare, hence many patients are already seriously ill at the time of presentation (7).

Quinti et al. followed, over a long period of time, 224 patients with CVID, with 75% of them under 14 years of age. The mean age at the time of diagnosis was 26.6 years. The mean age at the onset of symptoms was 16.9 years. This suggests a mean diagnostic delay of 8.9 years. Respiratory tract infections were the most prominent clinical problem (26).

Kidney problems are not commonly associated with CVID. An acute renal failure from IVIG therapy has been described as well as a nephrotic syndrome from secondary amyloidosis, a steroid-responsive nephrotic syndrome, and kidney dysfunction from granulomatous interstitial nephritis (27).

The clinical pathway of CVID in our patient was complicated with the development of HUS, which probably developed from a bacterial infection. The current outcomes of HUS have improved significantly with the use of plasma exchange, but the mortality remains unacceptably high at 10%–25% (17,28).

This is one of the rare cases of patients diagnosed with CVID which was complicated with HUS. The reason for the fatal outcome in our patient’s case was the belated CVID diagnosis with numerous complications—primarily the signs of advanced chronic pulmonary disease with consecutive cardiomyopathy.
